# Influence of Acquisition Time on MR Image Quality Estimated with Nonparametric Measures Based on Texture Features

**DOI:** 10.1155/2019/3706581

**Published:** 2019-11-20

**Authors:** Rafał Obuchowicz, Adam Piórkowski, Andrzej Urbanik, Michał Strzelecki

**Affiliations:** ^1^Department of Diagnostic Imaging, Jagiellonian University Medical College, Kraków 31-501, Poland; ^2^Department of Biocybernetics and Biomedical Engineering, AGH University of Science and Technology, Kraków 30-059, Poland; ^3^Institute of Electronics, Łódź University of Technology, Łódź 90-924, Poland

## Abstract

Correlation of parametrized image texture features (ITF) analyses conducted in different regions of interest (ROIs) overcomes limitations and reliably reflects image quality. The aim of this study is to propose a nonparametrical method and classify the quality of a magnetic resonance (MR) image that has undergone controlled degradation by using textural features in the image. Images of 41 patients, 17 women and 24 men, aged between 23 and 56 years were analyzed. T2-weighted sagittal sequences of the lumbar spine, cervical spine, and knee and T2-weighted coronal sequences of the shoulder and wrist were generated. The implementation of parallel imaging with the use of GRAPPA2, GRAPPA3, and GRAPPA4 led to a substantial reduction in the scanning time but also degraded image quality. The number of degraded image textural features was correlated with the scanning time. Longer scan times correlated with markedly higher ITF image persistence in comparison with images computed with reduced scan times. Higher ITF preservation was observed in images of bones in the spine and femur as compared to images of soft tissues, i.e., tendons and muscles. Finally, a nonparametrized image quality assessment based on an analysis of the ITF, computed for different tissues, correlating with the changes in acquisition time of the MR images, was successfully developed. The correlation between acquisition time and the number of reproducible features present in an MR image was found to yield the necessary assumptions to calculate the quality index.

## 1. Introduction

Magnetic resonance imaging (MRI) is the most universal medical imaging modality applicable to visualize soft tissues and bones. Although it has certain limitations, magnetic resonance (MR) yields a high degree of freedom to the image plane and sequence choice. This makes MR a widely used radiological modality in clinical radiology [1, 2]. However, MR image acquisition is a complex process. To obtain an MR image, the k-space has to be built in the phase-encoding direction, which collects the information on the position and amount of proton molecules present in the examined tissue. Not only is this step during image collection time consuming for medical staff and taxing to the patients, but it also generates large costs for healthcare providers [3]. Moreover, long periods of data collection are a possible source of motion artifacts associated with a decrease in the image quality [4]. This results in numerous call-back examinations and further reduces patient comfort and economic efficiency of the examination. Therefore, techniques that are able to reduce the scanning time are in high demand. One such technique is the parallel imaging technique, which relies on k-space undersampling realized by omitting parts of the lines in the phase-coding direction. As a result of the reduced amount of input data, reconstructed tissue images degrade to a lower quality [5]. Siemens has developed software with functionality, known as the I PAT, which provides accelerated image acquisition with a reduction in the number of echoes. This technique is known by its commercial name‐Generalized Autocalibrating Partially Parallel Acquisitions (GRAPPA), and is available for most imaging sequences [6–8]. GRAPPA1 can prevent undersampling. In GRAPPA2, half of the echoes are acquired with a 40% reduction in the signal. For GRAPPA3 and GRAPPA4, the time required to collate the image is reduced by 60 and 80%, in comparison with the original acquisition mode, respectively, despite a significant reduction in the acquired signal [9]. Existing measurements of image quality implemented in the system by vendors, such as the signal-to-noise ratio (SNR) and peak SNR (p-SNR), often do not reflect what medical professionals perceive from the image. Distortions of the imaged tissue, which affect the measurement, are a recognized factor of SNR measurement incompatibility [10–12]. These problems require a novel quality assessment technique for the MR image. Therefore, we focus on the concept of a technique for nonparametrized image quality assessment using an analysis of image features based on image textures, as it was performed for CT data [13]. Image texturing is one of the most important techniques used for object feature interpretation of an image. Image textures express complex visual patterns composed of entities, or subpatterns, which have a characteristic brightness, color, and size [14, 15]. Texture identification in an image can be regarded as grouping similar objects in an image to distribute the grey-level values among the neighboring pixels (for first-order textures) and in a given region of interest (for second-order textures) [16]. Textural features are used to parametrize the spatial patterns of an image responsible for its visual appearance as brightness coarseness, smoothness, and regularity, where most of these appearances are perceived by human senses [17]. Since the implementation of the MR analysis grey-level co-occurrence matrix by Haralick et al. [18], magnetic resonance image textures have been used for the analysis of medical imaging in certain cases, such as cerebral tissue [19–23], liver [24], breast [19], bone marrow [25], muscle [26], fat tissue [27], bone, the prostate for automated cancer detection [28], and colorectal cancer monitoring [29, 30]. The quality of diffusion tensor imaging (DTI) was also parametrized [31, 32]. Although these studies have outlined the possible implementation of image textural features (ITF) in clinical practice, as well as summarizing their anticipated usefulness in the analysis of different tissues, the application of these techniques to image quality measurements has not yet been proposed.

This study hypothesizes that image textures, which describe the properties of a visualized object if correlated with degraded images in a controlled manner and quantified, may precisely reflect image quality, which is a novel approach among MR image quality assessment methods. The proposed technique is accurate and repeatable, with the potential to compute the nonparametric MR image quality measures.

The aim of this study was to classify, in a nonparametric manner, the image quality of different tissues scanned under different conditions, with iterative degradation of the image quality with time.

## 2. Materials and Methods

**Figure 1:**
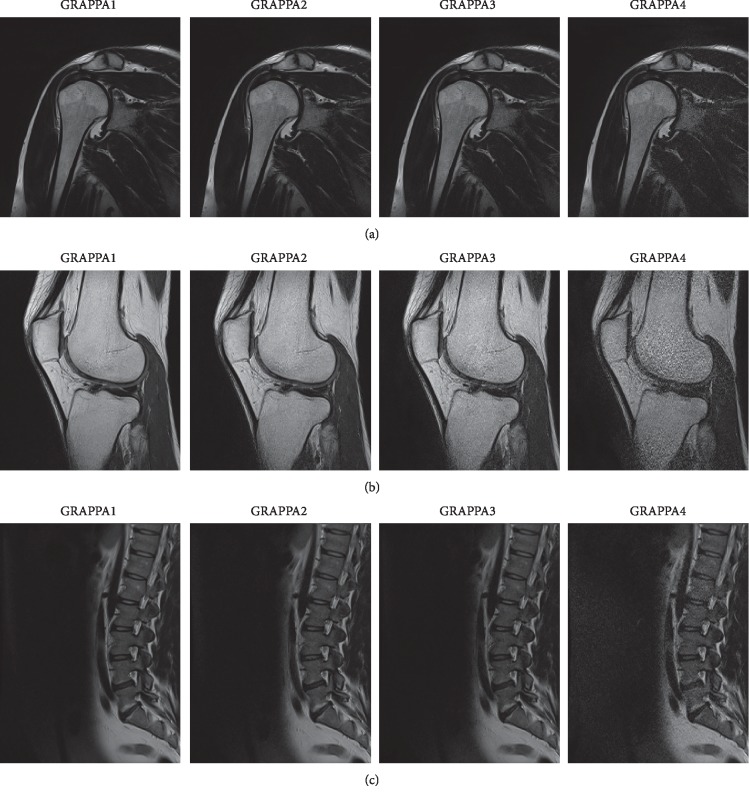
Changes in quality dependent on the GRAPPA method used. Hence, there were different acquisition times, for example, with (a) images of the shoulder, (b) images of the knee, and (c) images of the spine.

The study protocol was designed according to the guidelines of the Declaration of Helsinki and the Good Clinical Practice Declaration Statement. Special care was taken regarding personal data safety, where all images were anonymized before processing. Written acceptance to conduct this study was obtained from the Ethics Committee of Jagiellonian University (no.1072.6120.15.2017 dated: 20.06.2017). Data for 41 patients, 17 women and 24 men, between the ages of 23 and 56 years old, were utilized in the study. The criteria for negative selection were image artifacts that influenced image analysis. T2-weighted sagittal sequences of the lumbar spine and T2-weighted coronal sequences of the shoulder were analyzed (Figure 1). To routinely conduct MR studies of the spine and shoulder, shortened sequences, made using parallel imaging, were implemented with GRAPPA2, GRAPPA3, and GRAPPA4, with an average of 4 minutes added to the initial exam. For this study, we selected images of the tissues that were free of pathologies. A selection was made to meet stable (repetitive) conditions for texture feature analysis. Intrapatient variability strongly influenced texture parameter values by extending their range. Thus, a lack of correlation between the original and acquisition time (AT), reduced texture features, would have led to unclear conclusions, which can be caused by significant modifications of the image textures or large variability in evaluated feature values.

The MR DTI data were acquired from a 1.5 system Siemens Essenza (Erlangen, Germany) equipped with 12 dedicated table coils and 8 channel shoulder coils. With a gradient strength of 30 mT/m and a slew rate of 100 T/m/s, T2-weighted images were acquired. For the lumbar spine images, the following parameters were used: an applied echo time of 94 ms and a repetition time of 6,500 ms, with phase oversampling of 20, a distance factor of 30, and a flip angle of 150°. The scan geometry was as follows: an acquisition matrix of 143 × 256 and a slice thickness of 3 mm. A voxel of nonisotropic resolution at 0.8 × 0.8 × 3 mm was acquired.

For the shoulder coronal images, the following parameters were selected: an applied echo time of 101 ms and a repetition time of 3,540 ms, with a flip angle of 150°, a phase oversampling of 100, and a distance factor of 30. The scan geometry was as follows: an acquisition matrix of 256 × 320 and a slice thickness of 3 mm. A voxel of nonisotropic resolution at 0.8 × 0.6 × 3 mm was acquired.

For the knee sagittal images, the following parameters were selected: an applied echo time of 91 ms and a repetition time of 4,940 ms, with a flip angle of 150°, a phase oversampling of 63, and a distance factor of 30. The scan geometry was as follows: an acquisition matrix of 224 × 320 and a slice thickness of 3 mm. A voxel of nonisotropic resolution at 0.7 × 0.5 × 3 mm was acquired.

The images were sent to a dedicated PACS (Syngo Siemens). After anonymization, the images were postprocessed with dedicated indigenous software, i.e., MaZda 5.0–2012 (developed at the Technical University of Lodz, Institute of Electronics [33]), with the use of texture feature maps in the selected ROIs.

**Figure 2:**
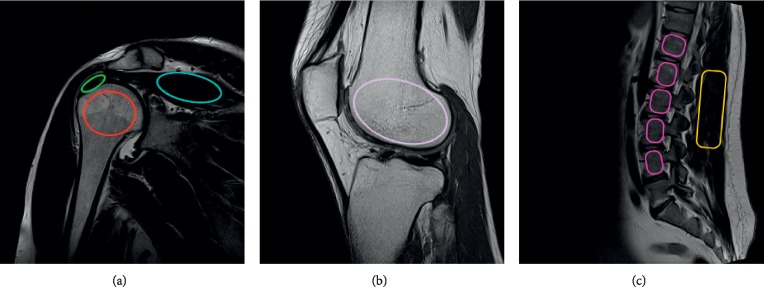
Analyzed ROI samples for different AT values for (a) humerus epiphysis (red), supraspinatus muscle (blue), and supraspinatus tendon (green), (b) femur epiphysis (pink), and (c) bone of the spine (violet) and muscles of the spine (yellow).

On three consecutive layers of each sequence (PAT and non-PAT), the ROI was visualized and analyzed for every patient (Figure 2). The average ROI sizes varied from 350 (tendon) to 1,150 (bone) pixels. Such ROI sizes are sufficiently large to represent the statistical textural features. The following 257 texture parameters were estimated and averaged for the ROI defined on three selected layers:

- Eleven grey-level co-occurrence matrix (GLCM) parameters [18]: such matrices were computed in four directions, i.e., vertical, horizontal, 45°, and 135°. In addition, five distances between the pixels, varying from 1 to 5, respectively, were considered. In total, 11 × 4 × 5 = 220 parameters were calculated.
- Five run-length matrix (RLM) parameters [18]: as for the co-occurrence matrices, the calculations included four directions, yielding 20 parameters. The run-length matrix elements, *R* [*i*, *j*], represent the number of pixel set occurrences of length *j* and brightness *i*.
- Five gradient matrix parameters: the image gradient matrix was initially determined with an appropriate filter using a 3 × 3 mask [18].
- Five parameters defined for the first-order autoregressive model (AR): this statistical model is based on the assumption that the brightness of a given image pixel depends on the weighted sum of the neighboring pixels [34].
- Sixteen parameters calculated based on the wavelet transformation (HW): these data represent the energy of the ROI subimages in the wavelet coefficient space. The wavelet transform was calculated using the Haar basis functions for four image scales, resulting in four subimages for each scale, which yielded 16 parameters in total [35].
- Nine first-order features (FOF) were histogram-based (mean, variance, skewness, kurtosis, and percentiles: 1, 10, 50, 90, and 99%, respectively).

Feature values of the texture maps were calculated for each patient. The results of the computations were further correlated with the reference images, with GRAPPA1 (no PAT images) and between the GRAPPA2–4 images, which were made with different parallel imaging algorithms associated with various reductions in the imaging time.

The following structures were selected and studied: bones of the spine (*n* = 8), extensor muscles of the back (*n* = 8), femur (*n* = 7), humerus epiphysis (*n* = 9), humerus metaphysis (*n* = 8), humerus shaft (*n* = 9), supraspinatus muscle (*n* = 9), and supraspinatus tendon (*n* = 9).

Next, following the methodology described by Midya et al. [36], Lin's concordance correlation coefficient (*ρ*_*c*_) was estimated between the reference textural features (obtained for reference images) and those estimated for the PAT images. In each analysis, sets of textural features (obtained for all patients, for which the given structures were visualized) were considered. Equation (1) defines  *ρ*_*c*_:

(1) $$\begin{array}{c} \rho_{c}=\frac{2\rho \sigma_{r}\sigma_{\text{PAT}}}{\sigma_{r}^{2}+\sigma_{\text{PAT}}^{2}+{\left(\mu_{r}-\mu_{\text{PAT}}\right)}^{2}}, \end{array}$$

where *μ* and  *σ*^2^ are the means and standard deviations of the reference and PAT texture feature vectors, respectively, and *ρ*  is a correlation coefficient between these two vectors. Features with *ρ*_*c*_ > 0.9 were considered to be reproducible, i.e., they preserve the properties of reference textures acquired for the PAT images. Furthermore, features with *ρ*_*c*_ > 0.8 were also recorded.

Finally, Lin's correlation coefficients for textural features averaged for all patients were estimated between the feature vector, which contains the values obtained for the reference, and three PAT images, as well as for the vector that consists of the normalized acquisition times. Features with *ρ*_*c*_ > 0.9 had a significant correlation with AT and can thus be used for image quality assessments.

## 3. Results

Based on Table 1, the largest number of textural features, which have a significant correlation between the reference and GRAPPA images, were obtained for the bones of the spine. For other structures, we observed a significant drop in these features. However, the number of highly correlated features was slightly higher for the femur. We note that the distribution of such features was not equal for all GRAPPA modes but the highest number of significant correlations was observed for AT2. For the humerus epiphysis and supraspinatus tendon, the number of reproducible textural features was insignificant. Highly correlated texture feature types are listed in the last column of Table 1 (best performing textural features). In nearly all cases, the GLCM features were characterized by high *ρ*_*c*_ (sum average, contrast). Another place in the set of highly correlated features was shared among those obtained from the RM matrix (short-run emphasis), the Haar wavelet parameters, and the first-order features (percentiles).

**Figure 3:**
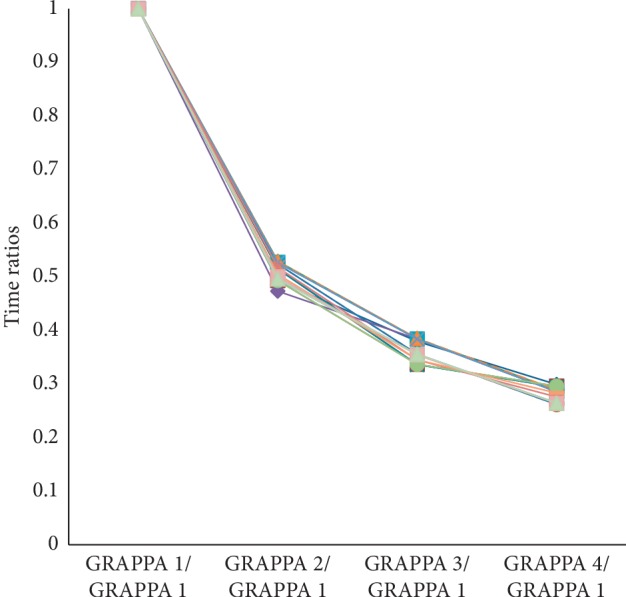
Correlations of different obtained acquisition times with various PATs (GRAPPA1–4). The *y*-axis represents the relative time computed by dividing the time required for examination with the use of GRAPPA1–4 by the time required for GRAPPA1 examination.

Different numbers of textural features that correlated with the acquisition times were obtained for the various (analyzed) structures. Feature values were estimated for four acquisition times, which were used as the relative ratio values (Figure 3). Figure 3 presents the relative time computed by dividing the time required for examination with the use of GRAPPA1–4 by the time required for GRAPPA1 examination. We note that time of these examinations was different due to different body masses of the patients but was dependent on the patient body mass and selected sequence.

Next, the values for each examination were averaged for all ROIs and patients. The average time for the femur and supraspinatus tendon was quite high while the average for the remaining structures was low, i.e., for the bones of the spine and humerus epiphysis.

## 4. Discussion

**Figure 4:**
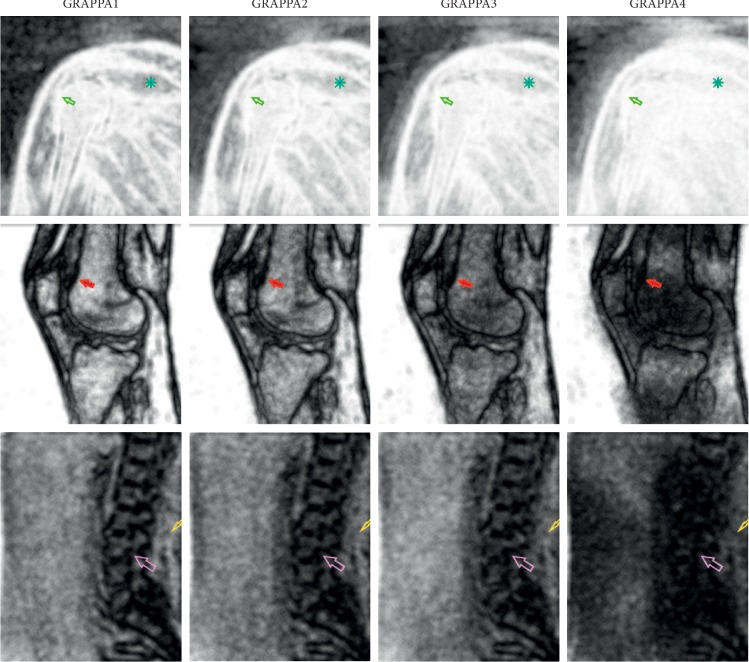
Texture feature maps (GLCM) of the MR images of the knee made with gradually reduced acquisition times (GRAPPA1–4). Image representation is shown on consecutive columns (left to right). The images show a gradual fading of the anatomical object features with a decreasing acquisition time. Note the degradation of the bone outline, which is a structure that is the most persistent on the image as indicated by arrows. The muscle tissue is indicated with an asterisk (upper row) and a small arrow (bottom row). The following texture features are present: GLCM entropy, histogram max, and GLCM-inverse difference moment.

The number of reproducible textural features depends on the signal-to-noise ratio (SNR) value of the particular structure. This parameter is one of the image quality measures, which can be estimated with the following equation:

(2) $$\begin{array}{c} \text{SNR}=\frac{\mu_{\text{ROI}}}{\sigma_{\text{noise}}^{2}}, \end{array}$$

where *μ*_ROI_ is the mean value intensity calculated for the ROI of the given structure and *σ*_noise_^2^ is the standard deviation of the noise, which was estimated as the standard deviation of the pixel intensities located in the image region with no signal (i.e., the top-left or right rectangle, with a size of 100 × 200 pixels, were selected for the estimation, depending on the image type).  Table 2 lists the average SNRs for all analyzed patients and estimates for the different structures. Based on Tables 1 and 2, the number of reproducible textural features correlates with the SNR. The reproducible textural features are high for larger SNRs (spine bones and femur) and low for structures that have a low SNR (i.e., tendons and muscles). This can be explained by the fact that a low SNR indicates a dominance of noise in the ROI and, thus, its texture characterizes the noise properties rather than the tissue itself. Therefore, there are no texture parameter correlations among the different PAT images as the texture modification caused by a shorter AT does not represent the changes in the tissue structure but reflects the varied noise distribution (Figure 4).

For structures with a high SNR, the texture describes the structure of the visualized tissue. Even for a shorter TA, certain textural features presented a good reproduction of the tissue components. We suggest that a large number of the reproducible textural features obtained by the ROI for the spine bone are actually caused by the high homogeneity of these textural features (i.e., the features are smoother and more homogeneous than the texture corresponding to the femur). Thus, the changes in the AT have a weaker influence on the spine bone texture when compared to rougher textures that characterize other analyzed tissues.

Thus far, other methods have been implemented to achieve the goal of this study. An extensively used method is the SNR, which is the quotient of the mean signal intensity distributed in the imaged object to the standard deviation of the noise, as it accounts for the background noise [11, 37]. Although popular, this method has several serious limitations. The manner in which it computes the quotient, i.e., by comparing the object and background noise with the object signal, renders it as an imprecise technique to estimate the noise of an examined object. Moreover, the SNR has other serious drawbacks, such as insensitivity to tissue-related artifacts [38]. Therefore, this method can underestimate patient-related artifacts provoked by magnetic field interference and gradient activity in the human tissue. Medical image readers also perceive the lack of accurately measured distortions caused by tissue-specific image degradation [38–42]. The fact that the SNR does not reflect differences in the perceptual image quality is a currently topic of debate [43–45]. The approach in this study circumvents these problems as image quality estimation is based on texture features in the given ROI, which does not require comparison with the signal bias in the reference points. Full quality assessment reference models are frequently used by the medical community, especially imaging professionals, such as radiologists. However, significant error plagues these perceptual techniques as a different medical personnel may observe different psychosomatic features. Furthermore, personnel may have different interpretation habits and may perform these observations in different environments (i.e., varying light conditions, display angles, and display distances) [43, 46]. Therefore, we are unable to compare the results of such interpretations.

Moreover, the SNR does not fully reflect the image quality in our study. First, the SNR value varies for different tissues in good quality images (GRAPPA 1). This is because the SNR is signal dependent (i.e., as a ratio of the ROI mean to the background standard deviation). For example, the SNR is high for the bone region (e.g., the humerus and vertebra) while it is low for muscles (see Table 2). The SNR is also characterized by high between-patient variability, as listed in Table 2, which lists the SNR standard deviations averaged for all patients. Only the SNR values for muscles (spine) are closely distributed around the mean value. Finally, reduction in the SNR for shorter acquisition times does not correspond to a loss of image quality. This can occur when mean SNR values are analyzed for different tissues in certain images in example supraspinatus-tendon (ST) and humerus-bone (HB) cases. For GRAPPA2 and GRAPPA3, significant differences between the SNR values were found due to the previously mentioned tissues. The mean SNR values recorded for the GRAPPA2 image was 10.59 and 131 for the ST and HB, respectively, and for the GRAPPA3 image, the SNR was 8 and 113.47 for the ST and HB, respectively.

To our knowledge, there are very few previous studies that have attempted to introduce nonparametric models for the successful quality estimation of MR images. The majority of previous studies focus on the complexity of quality analysis in different postprocessing modalities used in computed tomography (CT) associated with the use of postprocessing algorithms, such as SAFIRE, ADMIRE (Siemens), and ASiR (GE) [13, 36, 47, 48]. Woodard and Carley-Spencer [49] evaluated nonparametric measures of MR image quality, implementing an analysis of variance (ANOVA) to demonstrate the variation in different quality grades. This study also has several limitations concerning the restrictions of the ANOVA computational model, which affects quality measurement discrimination. Therefore, the proposition of a quality index computation based on the method of Woodard [49] is disputable.

Mortamet et al. [38] proposed another approach, applying the concept of a comparison background of the imaged tissue (scanned air area) and the tissue image itself. This approach is based on the assumption that the majority of the artifacts that appear in the imaged object propagate to the background, which inhibits a comparison. This technique is different from the current technique in terms of the features analysis of the selected object marked as an ROI. However, noise distribution estimation in Mortamet et al. [38] is similar to this study, which provided the assumption to propose a quality index.

Holli et al. [17] proposed an approach similar to the approach of this study, implementing first- and second-order statistics using MaZda 4.5 [33] to analyze the changes in the MR images of brain tissue of a subject suffering from mild head trauma. In contrast to this study, the primary objective of Holli et al. [17] was to present a plausible practical use for the ITF, but he was not concerned with image quality discrimination.

A study from Osadebey et al. [50] is the most similar one to our approach. Osadebey et al. [50] demonstrated a possible application of texture analysis to test local contrast and entropy as features of the image. Based on the MR images of brain tissue, Osadebey et al. [50] was able to propose a quality index. The use of the ITF combined with the demand to evaluate nonparametric measurements of MR image quality inspired this study to propose the current approach.

In this study, although we found a correlation between the ITF and acquisition times for different GRAPPA (1–4), several inconsistencies occurred in the results as improved correlations between the ITF and shorter scan times was found for GRAPPA3, which resulted in a reduced signal compared with GRAPPA2, where there was a significant improvement in signal preservation.

This inconsistency in the results can be explained by the impact of systematic errors and concomitant B-matrix uniformity that are present in the MR acquisitions. A recent investigation of the magnetic field's influence showed the existence and importance of systematic errors related to the use of hardware and the implementation of specific sequences [51–53]. There was a presence of concomitant field and eddy currents in the nonunity of the signal distribution generated by the gradient and RF coils, which resulted in B-matrix inhomogeneity [54]. These factors are, in part, responsible for the observed incompatibilities in this study, especially due to the use of sensitive echo-planar imaging (EPI) sequences.

The influence that the hardware (MRI scanner) has on image quality is important and cannot be neglected in the reconstruction algorithm. Although both cases yielded different signal losses, GRAPPA2 and GRAPPA3 produced a similar SNR quotient. Here, the execution of the reconstruction algorithm in both cases produces substantial uncontrolled noise [55]. In such cases, discriminating the features of the imaged object in the noisy time series is difficult, which was generally described by He [56] and specifically reviewed for CT images by Bielecka and Piórkowski [57]. The use of a larger series of images should overcome this issue, which will enable improved statistical discrimination with the possible evaluation of hardware-dependent differences. The novelty of this study is the evaluation of a comparative, multiparametric analysis for different ITFs computed at different ROIs derived from the various image qualities, which provided the assumption required to propose a numerical quality index. The major limitation of our study is the limited number of study samples. The analysis of different tissues using a larger number of samples is necessary to provide comprehensive and comparative results for different tissues and an evaluation of the reduction in time while maintaining image quality.

## 5. Conclusions

In this study, we proposed an approach to estimate image quality based on image texture analysis. As demonstrated in numerous previous studies, image texture correctly describes the properties of visualized organs and tissues. With a reduction in acquisition time, the values of certain texture features, estimated for the analyzed ROIs, changed and were no longer good descriptors of the analyzed tissues. This was caused by degraded image quality. A decreasing number of texture features indicates that the image of the visualized tissue was modified and does not resemble the original image. A reduction in the AT caused this effect, which is a known factor that influences image quality. Thus, we conclude that the number of texture features in a degraded image, which has a significant correlation with texture features in the original image, is an indicator of image quality. This, however, is a rather qualitative measure, and we are unable to estimate the number of correlating texture features that will yield good image quality. We can only state that larger numbers of such features improve the quality. However, we have demonstrated that the SNR does not fully reflect the image quality.

A benefit of our approach is that image quality assessment is based on the texture properties of the examined tissue. The texture describes a structure of the visualized object, and it is rather independent on the signal. Therefore, we assumed that the proposed approach is more objective because it can express object “fading” measured based on the decreased correlation of tissue textural parameters. The SNR is strongly dependent on the signal that in turn depends on acquisition protocol and scanner settings (thus, SNR is also user dependent). The proposed approach is less dependent on signal changes in different ROIs because by applying texture parameters, it considers variations in visualized object structure with degrading image quality.

Moreover, the SNR strongly depends on the signal contrary to applied texture analysis (i.e., instead texture represents tissue structure and not its intensity distribution).

The average values of the textural features correlate differently with the various acquisition times for different tissue samples. Therefore, nonparametric estimation of quality is possible for the ROI in the image but not in the entire scene or MR image. We consider quality assessments of pathological tissue images as an important issue that will be addressed in our future studies.

## Figures and Tables

**Table 1:** Number of correlated textures for the imaging sequences obtained with different acquisition times.

	GRAPPA1 vs. GRAPPA2		GRAPPA1 vs. GRAPPA3		GRAPPA1 vs. GRAPPA4		Feature values/acquisition times (F/A)	Best performing textural features
Correlation coefficient/tissue	>0.8	>0.9	>0.8	>0.9	>0.8	>0.9	>0.9	
Bones of the spine (8 patients)	212	166	185	141	60	49	9	GLCM, RLM, Haar wavelets
Extensor muscles (8 patients)	1	0	1	0	0	0	71	GLCM, FOF
Femur (7 patients)	14	8	9	5	6	4	114	GLCM, RLM, Haar wavelets
Humerus shaft (9 patients)	1	1	9	5	0	0	39	FOF, GLCM
Humerus epiphysis (9 patients)	0	0	6	1	0	0	3	FOF, GLCM
Humerus metaphysis (9 patients)	0	0	10	2	0	0	18	RLM, Haar wavelets
Supraspinatus muscle (9 patients)	0	0	12	0	1	0	42	GLCM, Haar wavelets
Supraspinatus tendon (9 patients)	0	0	3	0	0	0	108	GLCM

Results are listed for the different scanned tissues. F/A indicates the features acquired and the correlation with different acquisition times.

**Table 2:** Mean values and standard deviations of the SNR estimated for different structures.

ROI	GRAPPA1 SNR	
	Mean	Standard deviation
Tendon	14.47	11.95
Humerus	172.48	143.70
Muscle (shoulder)	10.31	1.59
Vertebra	131.97	161.83
Muscles (spine)	51.29	48.60

## Data Availability

The image data used to support the findings of this study were supplied by Jagiellonian University under license and, therefore, cannot be made freely available. Requests for access to these data should be addressed to the corresponding author.
